# PEC‐DFM Hybrid‐Sensor Based on Nanoparticle‐Resolved Estimation for Sensitive and Reversible Glucose Monitoring

**DOI:** 10.1002/advs.202523818

**Published:** 2026-03-24

**Authors:** Zihao Zhang, Junzheng Liu, Qinggong Wu, Yuan Zeng, Gang Xiao, Zhao Yue, Zhigang Zang, Shuang Zhao

**Affiliations:** ^1^ Key Laboratory for Special Fiber and Fiber Sensor of Hebei Province School of Information Science and Engineering Yanshan University Qinhuangdao P. R. China; ^2^ College of New Materials and New Energies Shenzhen Technology University Shenzhen P. R. China; ^3^ Department of Microelectronics Nankai University Tianjin P. R. China; ^4^ College of Optoelectronic Engineering Chongqing University Chongqing P. R. China

**Keywords:** reversible sensing mechanism, PEC‐DFM hybrid‐sensing system, photoelectrochemical sensor, ultrasensitive and reversible glucose detection

## Abstract

The accurate detection of glucose in wound exudate is critically important for monitoring chronic wound healing. While photoelectrochemical (PEC) sensors are widely used, they often suffer from material degradation caused by redox reactions during operation. Herein, we introduce a dark‐field microscopy (DFM) setup integrated with a PEC system, creating a PEC‐DFM hybrid‐sensing platform. This system allows for conjoint measurement of photocurrent and monitoring of nanoparticle behavior via scattering spectroscopy. We further introduce Ag NP@Au NCs nanocomposites to drastically improve sensor performance, achieving a 193‐fold increase in signal‐to‐noise ratio. The PEC‐DFM hybrid‐sensing system guides the optimization of the nanocomposite's structure, enabling exclusion of damaged nanoparticles during measurement. Moreover, through continuous, long‐term (up to 1800 s) observation of scattering spectrum parameters, we decipher the photocurrent generation mechanism, confirming electron transfer from Au NCs to Ag NP inside single Ag NP@Au NCs nanocomposite. Finally, a non‐invasive sensing mechanism based on molecular‐level competition for glucose is developed, enabling reversible (20 rounds) and ultrasensitive detection of glucose with a detection limit of 0.49 p **m**. This work not only provides a powerful tool for wound management but also offers profound insights into the design of advanced hybrid sensing systems.

## Graphical abstract

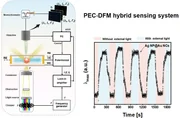

## 1 Introduction

Glucose is a critical biomarker that can be detected from wound exudate for evaluating chronic wound healing status and guiding timely clinical intervention [1]. In comparison with other biomarkers, glucose exhibits high biocompatibility [2] and direct correlations with metabolism along with infection status [3], particularly in patients with diabetes [4]. However, the accurate detection of glucose in wound exudate remains a long‐standing challenge clinically. Its primary obstacle stems from the inherent disadvantages of wound exudate such as small volume (typically microliter‐scale) and limited glucose level [4, 5]. For this purpose, sensitive detection is essential to capture low‐abundance analytes with limited amounts, and reversibility is critical enabling the sensor to be reused for multiple detections and real‐time monitoring, which reduces the consumption of small‐volume samples and improves the reliability of the sensing processes [6]. Thus, this necessitates that the measurement be completed with ultrahigh sensitivity and excellent reversibility.

Traditional glucose detection technologies are primarily optical ones (such as surface plasmon resonance [7], colorimetry [8], fluorescence [9]) and electrical ones (such as electrochemistry [10] and photoelectrochemistry [11]). Among them, the optical ones typically exhibit ultrasensitive performance as 0.1 µm level limit of detection (LOD) [8]. The electrochemical (EC) approaches achieve impressively low limits of detection, reaching the 2 nm level with remarkable precision [12], and it holds great potential to be expanded into multiplexed sensing platforms, capable of simultaneously detecting multiple analytes through sensing arrays [13]. However, both methods employ the same signal type for input and output (either optical or electrical) during the sensing processes, leading to potential interference between the output signal and the input signal, which degrades the signal‐to‐noise ratio (SNR) [14]. For photoelectrochemical (PEC) systems, enhanced sensing performance can be achieved by employing light as the input signal (optical) and photocurrent as the output signal (electrical). The distinct nature of these signals minimizes interference, thereby enabling an excellent SNR and low LOD. To date, for example, a LOD as low as 0.33 nm can be achieved using nickel oxide nanosheets as photoelectrodes in a PEC system [15]. Thus, a highly sensitive glucose sensor can be fabricated via a PEC platform.

However, the most used sensing mechanism of PEC system is based on redox reactions using light‐triggered electrons transferring between nanomaterials and the electrolytes [16]. This may lead to structural damage of nanomaterials, particularly catalytic ones such as commonly used silver nanoparticles, resulting in unstable photocurrent and poor‐ reversibility [17]. Therefore, the monitoring of nanomaterials in PEC systems is essential. However, this remains highly challenging because of the uncleared PEC working mechanism, single nanoparticle level spatial resolution, dynamic tracking capability and signal‐to‐noise ratio [18, 19]. To address this issue, PEC systems can be integrated with complementary microscopy to construct hybrid sensing platforms, such as PEC‐Raman scattering microscopy hybrid‐sensing system (monitoring of redox processes) [20] and the integration of confocal fluorescence microscopy (in vivo monitoring) [21]. Unfortunately, these systems monitor entire electrodes rather than individual nanoparticles. Dark‐field microscopy (DFM), as a powerful technique, can leverage the light scattered by metallic nanoparticles to image single nanoparticle with high spatial resolution, enabling mechanism study and dynamic tracking behaviors of single nanoparticle [22]. DFM system has high SNR because all direct and unscattered illumination is removed and only light scattered by nanoparticles is collected, creating a near‐zero dark background [19]. To date, DFM systems have demonstrated the capability to continuously image and monitor individual gold nanoparticles and single proteins for up to 24 h [23]. Therefore, PEC‐DFM hybrid‐sensing system can be built to monitor nanomaterials in PEC systems to obtain stable photocurrent, establishing a necessary foundation for reversible glucose detection. A reversible sensor can be featured by reversible interactions (such as redox reversible reactions, chemical reversible binding and biological reversible binding) between analytes and sensing elements [24]. As shown in Table S2, most of these sensors are designed to detect redox‐active species including metal ions, gaseous analytes, and small inorganic molecules. New sensing mechanism should be explored focusing on non‐redox molecules. Based on the discussion above, by using PEC‐DFM hybrid‐sensing system, the stability of photocurrent can be enhanced, and together with a reversible sensing mechanism, a reversible sensor for non‐redox small molecules such as glucose can be realized.

The integration of a PEC system with a DFM setup has few reports recently. A major challenge in DFM‐based PEC sensing systems lies in the small photocurrent amplitude generated by the PEC system when sharing a light source with DFM in which the illumination intensity is typically low. A feasible strategy to overcome this limitation involves the selection of appropriate nanomaterials for use in the PEC system. Recently, an increasing variety of nanomaterials have been employed in PEC sensors, including quantum dots (QDs) [25], carbon dots (CDs) [26], metal nanoclusters (NCs) [27], halide perovskites (HPs) [28] and rare earth‐based materials [14]. Among these, metal NCs offer distinct advantages such as excellent biocompatibility and superior catalytic activity, contributing to outstanding PEC performance [27]. Recent years, metal NC‐based nanocomposites have become a prevailing trend in PEC systems, exemplified by Ag/Au NC/BiVO_4_ [29] and Au NC/TiO_2_ electrodes [30]. Metal nanoparticles can be effective supports for enhancing PEC performance through hot electrons via localized surface plasmon resonance (LSPR) [31]. Given their strong catalytic properties and low‐cost, silver nanoparticles show particular promise among noble metal nanoparticles in PEC applications. For example, using an Ag/CeO_2_ core/shell nanostructure, the Ag core enhances the PEC performance of the CeO_2_ shell by a factor of 50 [14]. Meanwhile, silver nanoparticles also have been widely applied in the DFM system, and the use of silver nanoparticles is favorable for the conjoint measurements of PEC and DFM systems. Advances in this field have already facilitated the study of Ag nanoparticles as small as 10 nm (Table S1) [32]. Thus, in this paper, silver nanoparticle@gold nanoclusters (Ag NP@Au NCs) nanocomposites are introduced to improve the photocurrent amplitude and sensitivity of the sensor.

This study presents a reversible glucose sensor based on Ag NP@Au NCs hybrid nanocomposites to enhance the photocurrent amplitude and sensitivity. A DFM system is integrated into the PEC system to construct a PEC‐DFM hybrid sensing platform. This design not only uncovers the working mechanism of the PEC system, but also excludes nanoparticle damaged, thereby improving the photocurrent stability which is critical for achieving reversible detection. Moreover, a reversible sensing mechanism specially for non‐redox small molecules is given and explored. Consequently, owing to the enhanced sensitivity and stability, the sensor enables reliable glucose detection in wound exudate.

## 2 Results and Discussion

### 2.1 Characterizations of Nanomaterial and Working Electrode

**FIGURE 1:**
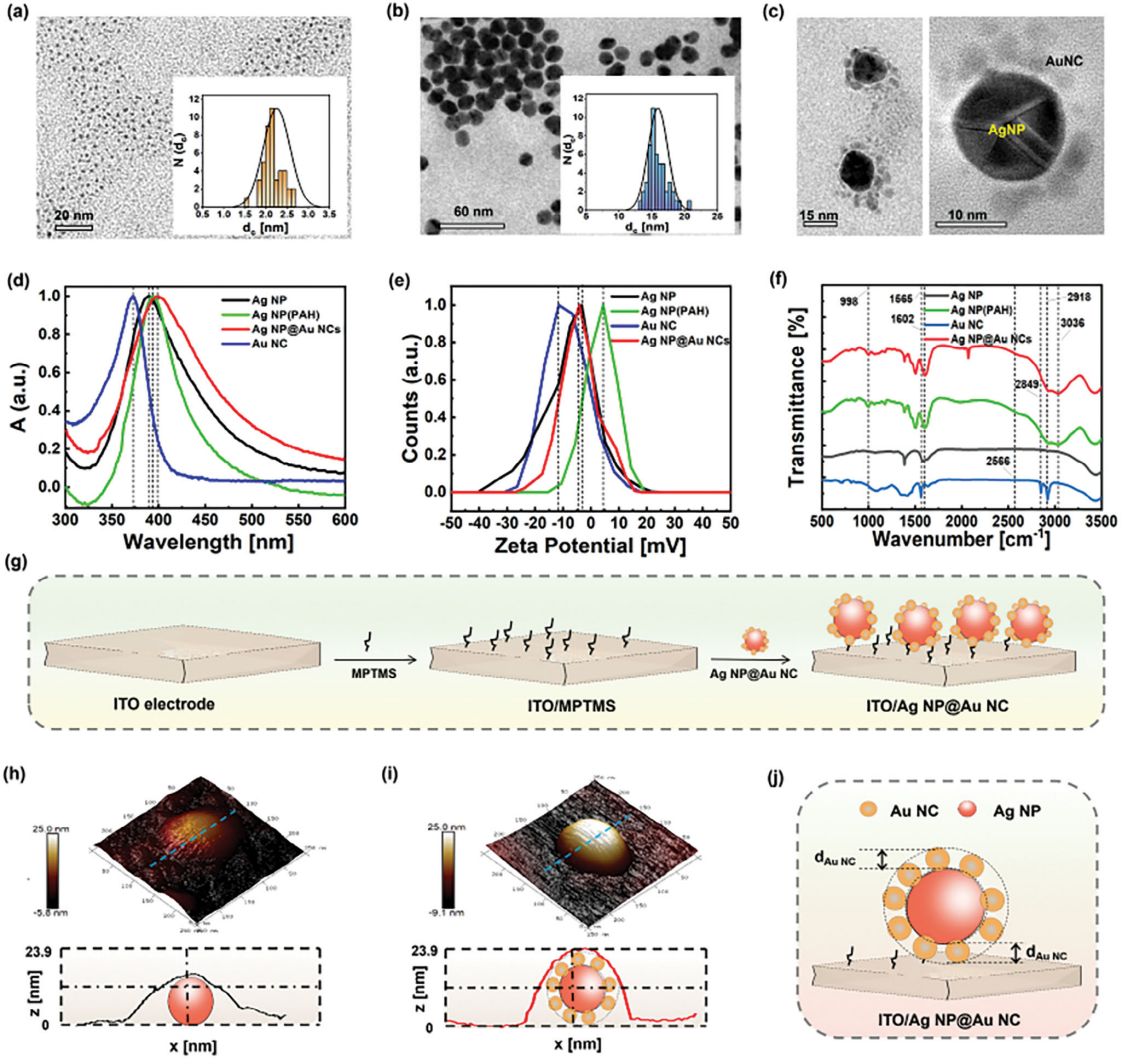
TEM image and its size (*d*_c_) distribution diagram of (a) Au NCs and (b) Ag NP. (c) HTEM image of Ag NP@Au NCs nanocomposite (*d*_c_ of Ag NP is 15.7 nm). Results from (d) UV–vis absorption spectroscopy, (e) Zeta potential (*ζ*) measurements and (f) FTIR spectroscopy of Au NCs, Ag NP, Ag NP(PAH) and Ag NP@Au NCs. (g) Schematic diagram of self‐assembly of nanomaterials on working electrodes. AFM measurements from individual (h) Ag NP and (i) Ag NP@Au NCs nanocomposite immobilized on the indium tin oxide (ITO) working electrode. The height profile is along the plotted blue line. (j) The graphic illustration of Ag NP@Au NCs immobilized on working electrode.

Au NCs are synthesized using 11‐mercaptoundecanoic acid (MUA) as a capping ligand, following previously established protocols with minor modifications [33, 34]. The bandgap of MUA‐Au NCs is estimated to be 3.1 eV through Tauc plot analysis combined with Mott‐Schottky measurements (see calculations in Section II of the Supporting Information). This value can be tuned by modifying the surface ligands, highlighting a key advantage for the flexible design of PEC sensors. Additionally, the MUA ligand effectively prevents aggregation and optical quenching, thereby ensuring excellent stability of the PEC sensor. In Figure 1a, the synthesized Au NCs are characterized by transmission electron microscopy (TEM), which reveals good dispersion and an average diameter (*d*_c_) of 2.1 ± 0.2 nm. This  can be further supported by atomic force microscopy (AFM) results after immobilization of Au NCs on the working electrode (Figure S6), where a height of 3.65 nm is observed and slightly larger than the TEM measurement due to the presence of surface organic ligands that are not detectable by TEM. The resulting Au NCs are subsequently used to construct Ag NP@Au NCs nanocomposites.

According to the literature [35], Ag NPs are synthesized, and their size is characterized by TEM (Figure 1b), yielding an average diameter of 15.7 ± 1.5 nm. The particles exhibit uniform spherical morphology, well‐defined edges, and a narrow size distribution, with no observable aggregation. These Ag NPs are then employed to anchor Au NCs for the formation of Ag NP@Au NCs nanocomposites, following a reported method with slight modifications [36]. The resulting Ag NP@Au NCs nanocomposite is characterized by high‐resolution transmission electron microscopy (HRTEM), as shown in Figure 1c (*d*_c_ = 20.4 ± 1.7 nm), clearly revealing a central Ag NP core surrounded by Au NCs. Notably, the Ag NP is not fully encapsulated by Au NCs, leaving exposed regions that can directly interact with incident light for LSPR. Consequently, the embedded Ag NP within the Ag NP@Au NCs nanocomposite can be simultaneously excited during both PEC and DFM measurements.

The formation of the Ag NP@Au NCs nanostructure is further investigated using UV–vis absorption spectroscopy and zeta potential measurements, as shown in Figure 1d,e, respectively. The Ag NPs are synthesized via citrate reduction, resulting in a negatively charged surface (ζ_Ag NP_ = −2.9 mV), which is confirmed by zeta potential analysis in Figure 1e. Based on this, a positively charged polyelectrolyte, poly(allylamine hydrochloride) (PAH), can be adsorbed onto the Ag NP surface through electrostatic interactions to form Ag NP(PAH) in solution, facilitated by appropriate pKa values [37]. A schematic illustration of this process is provided in Figure S1. The Ag NP(PAH) exhibits a slightly larger size than the plain Ag NP, as evidenced by a redshifted UV–vis absorption spectrum relative to the bare Ag NP (Figure 1d). Furthermore, zeta potential characterization (Figure 1e) reveals an increased surface potential for Ag NP(PAH) (ζ_Ag NP(PAH)_ = +4.2 mV) compared to the original Ag NP, with ζ_Ag NP_ < ζ_Ag NP(PAH)_, confirming the presence of PAH on the nanoparticle surface. Subsequently, negatively charged Au NCs (ζ_Au NC_ = −11.5 mV), functionalized with –COOH groups from MUA ligands, are conjugated onto the Ag NP(PAH) surface via electrostatic attraction. As shown in Figure 1d, a distinct redshift in the absorption spectrum of the Ag NP@Au NCs nanocomposite relative to Ag NP(PAH) supports successful assembly. Moreover, the surface potential of the Ag NP@Au NCs nanocomposite reverts to negative (ζ_Ag NP@Au NCs_ = −4.6 mV), with ζ_Ag NP@Au NCs_ < ζ_Ag NP(PAH)_, indicating the successful formation of the Ag NP@Au NCs nanocomposite in solution.

Additionally, the successful synthesis of the Ag NP@Au NCs nanocomposite is verified by Fourier transform infrared (FTIR) spectroscopy (Figure 1f). For the Au NC spectrum, the peak at 1565 cm^−^^1^ corresponds to the stretching vibration of the C═O bond in the –COOH group of MUA ligands, while peaks at 2849 cm^−^^1^ and 2918 cm^−^^1^ are attributed to C–H stretching vibrations, confirming the presence of MUA [38]. According to the literature [39], signals around 2566 cm^−^^1^ originate from –SH groups. However, no such peak is observed in the Au NC spectrum, indicating complete coordination of MUA ligands to the Au atoms within the cluster, thereby confirming successful synthesis of MUA‐capped Au NCs. In the case of Ag NP(PAH), characteristic peaks at 998 cm^−^^1^, 1602 cm^−^^1^, and 3036 cm^−^^1^ appear in comparison to bare Ag NPs, which are assigned to the CH_2_–NH_3_^+^ symmetric stretch, N–H bending, and –NH_3_^+^ asymmetric stretch of PAH, respectively [40]. This confirms successful surface modification of Ag NPs with PAH. For the Ag NP@Au NCs nanocomposite, a notable decrease in the intensity of the 998 cm^−^^1^ peak is observed compared to Ag NP(PAH), which can be attributed to the electrostatic interaction between the –COO^−^ groups of Au NCs and the CH_2_–NH_3_^+^ moieties of PAH [41]. These results demonstrate that Ag NPs and Au NCs are linked through electrostatic adsorption mediated by the PAH polyelectrolyte and MUA ligands.

Furthermore, the Ag NP@Au NCs nanocomposite is characterized by dynamic light scattering (DLS) (Figures S8 and S9). Comparing with the plain Ag NPs in Figure S9, the hydrodynamic diameters (*d*_h_) of Au NCs and Ag NPs are measured as 6.1 nm (Figure S8a) and 15.7 nm (Figure S8b), respectively. The Ag NP@Au NCs nanocomposite exhibits a *d*_h_ of 27.8 nm (Figure S8c), approximately equal to the sum of the *d*_h_ of Ag NP and twice the *d*_h_ of Au NCs, suggesting that the Ag NP is surrounded by a monolayer of Au NCs, as illustrated in Figure 1j. It should be noted that DLS measures hydrated particle size, whereas TEM cannot resolve low‐electron‐density organic layers. Therefore, although the size trends are consistent, the d_h_ values are slightly larger than the d_c_ values obtained from TEM. These results confirm the successful attachment of Au NCs onto the Ag NP surface, forming the Ag NP@Au NCs nanocomposite as discussed above.

Finally, the Ag NP@Au NCs nanocomposite is analyzed by atomic force microscopy (AFM) after immobilization on indium tin oxide (ITO) substrates via a self‐assembly method (Figure 1g). The hydroxylated surface of the working electrode undergoes silylation with (3‐mercaptopropyl)trimethoxysilane (MPTMS), generating exposed –SH groups suitable for binding Ag NP@Au NCs [42] (Figure S1). In Figure 1h, AFM imaging of individual Ag NPs reveals a height (*h*_Ag NP_) of 16.6 nm, closely matching the TEM‐derived d_c_ value of 15.7 ± 1.5 nm (Figure 1b), confirming successful immobilization. Similarly, the AFM image of Ag NP@Au NCs (Figure 1i) shows a height of 23.9 nm (*h*_Ag NP@Au NCs_), consistent with the TEM *d*_c_ of 20.4 ± 1.7 nm (Figure 1c). Moreover, the AgNP@AuNCs/ITO working electrode can be characterized and calculated according to reported literature verifying that the aggregation of AgNP@AuNCs nanocomposites can be neglected [36, 43]. Based on these measurements and as schematically illustrated in Figure 1j, the thickness of the surrounding Au NC layer (*h*_Au NC_) is calculated as (*h*_Ag NP@Au NCs_‐ *h*_Ag NP_)/2 = 3.65 nm. This value aligns well with both the TEM data (Figure 1a) and AFM results for Au NCs immobilized on the electrode (Figure S6), with the slight increase attributable to the presence of surface organic ligands. Thus, both the successful synthesis and effective immobilization of the Ag NP@Au NCs nanocomposite are experimentally validated.

### 2.2 PEC‐DFM Hybrid‐Sensing System for Ag NP@Au NCs Nanocomposite Measurement

A PEC system is used in this paper to investigate the properties of nanomaterials and completing sensing applications because of its high signal‐to‐noise ratio (SNR). Based on the inherent advantage of single‐nanoparticle analysis of dark‐field microscope (DFM), DFM is introduced as assistance to the PEC system to develop a PEC‐DFM hybrid system for two main purposes. First, DFM serves to quantify the number of Ag NPs on the WE of PEC system, eliminate Ag NPs in suboptimal states, and enable a more accurate estimation of photocurrent values, thereby facilitating a better understanding of the performance of nanomaterials. Second, the DFM can help to conduct mechanism study of photocurrent generation based on the scattering spectra from single Ag NP in DFM system. It should be noticed that the result from PEC is never photocurrent from single nanoparticle but values calculated by averaging photocurrent per nanoparticle. The details of the PEC‐DFM hybrid system are shown as follows.

**FIGURE 2:**
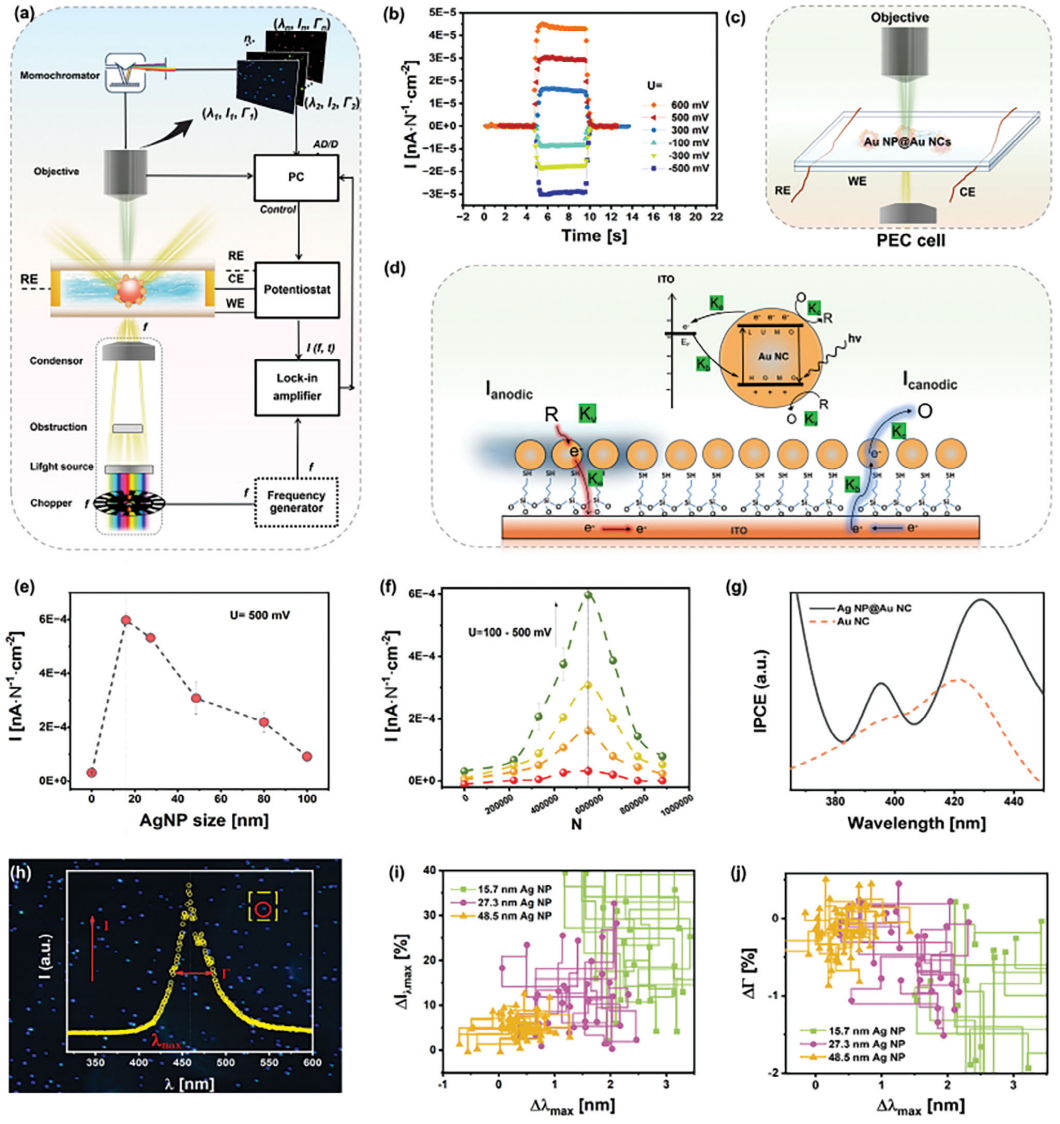
(a) Schematic illustration of home‐built PEC‐DFM hybrid‐sensing system for PEC measurement of individual Ag NP@Au NCs. White light is directed to Ag NP@Au NCs sample observing Ag NP number (*n*_Ag NP_) immobilized on working electrode by an objective and CCD together with scattering spectra collected to filter Ag NP which are undamaged. The PEC results of photocurrent (*I*_Ag NP@Au NCs_) are obtained from the sample in 0.01 M PBS (pH = 7.4) under potentials (*U*) applied on WE. With the help of a home‐built software, the final photocurrent output is the absolute value of *I* = *I*_Ag NP@Au NCs_/*n*_Ag NP_ where the *n*_Ag NP_ is obtained from the CCD output based on the image recognition technique (Figure S1). (b) Photocurrent behavior per area from Au NCs immobilized on the working electrode in 0.01 m PBS (pH = 7.4). (c) The PEC cell containing three‐electrodes including CE, RE and WE based on 0.7 mm ITO glass and 0.3 mm parafilm. (d) The graphic illustration of photocurrent generation from Au NCs. (e) The Ag NP size dependence from photocurrent per area based on Ag NP@Au NCs nanocomposite in PBS at 500 mV. The Ag NP number (*N* = *n*_Ag NP_ = *n*_Ag NP@Au NCs_ = 550708) and Au NCs amounts on each Ag NP are controlled equal for comparison calculated in section I of Supporting Information. (f) The number (*N* = n_Ag NP@Au NCs_) dependence from photocurrent (*I*) per area using Ag NP@Au NCs nanocomposite (*d*_c_ = 15.7 nm Ag NP) (*U* = 100–500 mV). (g) The incident photon‐to‐electron conversion efficiency (IPCE) results from Ag NP@Au NCs and plain Au NCs. (h) Scattering spectrum from individual Ag NP@Au NCs nanocomposites (*d*_c_ = 15.7 nm Ag NP) to output *λ*_max_, *I_λ_*_max_, *Γ*. The background is CCD image from individual Ag NP@Au NCs using image recognition technique to output *n*_Ag NP_. Using the baseline from the internal light excitation of DFM, the Ag NP@Au NCs characterized by DFM revealing the change of scattering spectrum as (i) Δ*I*_max_‐Δ*λ*_max_ and (j) ΔΓ‐Δ*λ*_max_ when an extra external excitation light is applied.

The PEC‐DFM hybrid‐sensing system built is presented as shown in Figure 2a and overviewed as follows. It is comprised primarily of a potentiostat with three‐electrode system, a lock‐in amplifier, a frequency generator, a Xenon white light source, an AD/DA converter, a computer, a dark‐field microscope (DFM) and a cell used for PEC measurement and scattering spectrum collection. The potentiostat is connected to the homemade PEC cell through three‐electrode system, namely the Pt reference electrode (RE), Pt counter electrode (CE) and ITO working electrode (WE). With the help of a frequency generator (*f* = 71 Hz) and an optical chopper, the frequency of the photocurrent from Ag NP@Au NCs nanocomposite is modulated for signal‐to‐noise ratio (SNR) improvement in the lock‐in amplifier. Afterwards, the processed photocurrent (*I*_Ag NP@Au NCs_) will be input to PC and collected by a software homebuilt. At the same time, the light spots scattered by the Ag NP@Au NCs nanocomposites can be collected by the CCD camera of DFM using a long exposure larger than 1 s due to the low quantum efficiency of small size of Ag NP. It should be noticed that the scattered light is from the Ag NP part in Ag NP@Au NCs nanocomposites, thus the number of Ag NP@Au NCs (*n*_Ag NP@Au NCs_) can be reflected by the light spots of Ag NP with the help of image recognition technique. Additionally, the scattering light from each individual Ag NP@Au NCs can be collected and then output into a monochromator. By analyzing the light intensity of each wavelength, a scattering spectrum can be formed as shown in Figure 2h. Three parameters of *λ*_max_ (peak resonance wavelength), *I* (intensity of the spectrum at *λ*_max_) and *Γ* (fullwidth at the half‐maximum of the spectrum) extracted from the spectrum can be continuously collected in real‐time as long as 1800s. The photocurrent generation in PEC system is based on electron loss or gain of redox reactions, which might damage the working electrode, so that the monitoring of Ag NP@Au NCs nanocomposites using PEC‐DFM hybrid‐sensing system is essential. The Ag NP damaged can be judged and excluded from the *n*_Ag NP@Au NCs_ with the help of *λ*_max_, *I_λ_*_max_ and *Γ*. The method of excluding the damaged nanoparticle can be found in Figure S2. The computer oversees the whole operations including the PEC measurement (*I*_Ag NP@Au NCs_) and the scattering spectrum acquisition (*n*_Ag NP_, *λ*_max_, *I_λ_*_max_, *Γ*) as shown in Figure S2c, and final photocurrent can be generated (*I* = *I*_Ag NP@Au NCs_/*n*_Ag NP@Au NCs_). The PEC‐DFM hybrid‐sensing system can exclude the Ag NP@Au NCs damaged and will be used for further measurements.

The usability of PEC‐DFM hybrid‐sensing system is initially verified through the PEC measurements of Au NCs self‐assembled on ITO working electrode by MPTMS. In Figure 2b, the photocurrent from plain Au NCs can be collected when different potentials (*U*) are applied. The *U*‐dependent and stable photocurrent output in Figure 2c verify the practicality of the PEC system and the PEC cell. This cell shown in Figure 2c can be used for conjoint measurements of PEC and DFM. The illustration of photocurrent generation mechanism from Au NCs on ITO can be given in Figure 2d. In detail, when there is no light, the insulated MPTMS layer under Au NCs will prevent electron transfer between Au NCs and ITO (process of *K*_b_ or *K*_e_), thereby observing zero photocurrent. When light is applied, electron–hole pairs are generated inside Au NCs at highest occupied molecular orbital (HOMO) and lowest unoccupied molecular orbital (LUMO), accompanying the recombination of electron–hole pairs and the emission of fluorescence. These charged carriers will exchange with the ITO working electrode by tunneling processes (*K*_b_ or *K*_e_) through the insulating MPTMS layer. *K*_b_ or *K*_e_ is affected by the barrier height between the Fermi level of ITO and the position of HOMO and LUMO. The Fermi level of ITO depends on the *U* applied on the working electrode. The HOMO and LUMO of Au NCs are determined by several key aspects such as the ligands and the atom structure of the Au NCs. Thus, when the *U* is determined, the final photocurrent of Au NCs is based on the competition of *K*_b_ and *K*_e_. When *K*_b_>*K*e, cathodic photocurrent can be collected, and on the contrary, anodic photocurrent can be obtained. Based on the discussion above, the effective PEC measurement in the PEC‐DFM hybrid‐sensing system is confirmed.

Afterwards, in order to meet the sensing performance requirements, the signal‐to‐noise ratio (SNR) of the PEC system is further improved by increasing the photocurrent amplitude. First, Ag NP@Au NCs nanocomposites are introduced in this work to obtain improved photocurrent from Ag NP@Au NCs by local surface plasmon resonance (LSPR) from Ag NPs. From Figure 2e, the photocurrent from Au NCs is increased 20 times by the Ag NP@Au NCs at maximum (*d*_c_ = 15.7 nm). This is reasonable due to the charged carrier recombination in Au NCs is limited by the hot electrons from Ag NP generated by the LSPR [14]. Second, the size dependence of Ag NP in the Ag NP@Au NCs nanocomposites is studied in Figure 2e. Five types of Ag NPs including 15.7 ± 1.5, 27.3 ± 2.3, 48.5 ± 6.3, 80.5 ± 5.1, 100.3±7.2 nm are synthesized according literature with minor modifications [35] and their sizes are characterized by DLS in Figure S9. When Au NCs are connected to Ag NP as the graphic illustration shown in Figure S1, the Ag NP@Au NCs reveals enlarged size than plain Ag NP due to the Au NCs surrounded using DLS characterization (Figure S8). To study the Ag NP size effect, Au NCs number (*n*_Au NC_) on each Ag NP is equally controlled as well as the Ag NP number (*n*_Ag NP_) on the ITO (calculated in Figure S1). In Figure 2e, as Ag NP size decreases, the photocurrent from Ag NP@Au NCs nanocomposites increases revealing the optimal photocurrent obtained from the Ag NP of *d*_c_ = 15.7 nm. Similar results are observed from previous work using Au NP@CeO_2_ and Ag NP@CeO_2_ core@shell hybrid nanoparticles in PEC system [14, 43]. Reasonable explanation can be given that smaller Ag NP has smaller surface area but higher energy of hot electrons [11]. Third, the number (*n*_Ag NP@Au NCs_) dependence of Ag NP@Au NCs from photocurrent (*I*) is studied at varied *U* applied from 100 to 500 mV versus reference electrode in Figure 2f. At the beginning, as the number increases, the photocurrent increases dramatically until the *n*_Ag NP@Au NCs_ of peaks reaches around 550 708 achieving a 193‐fold increase in signal‐to‐noise ratio (SNR) (SNR = photocurrent/dark current). The detailed calculation process can be found in Section I of the Supporting Information. This is due to more light‐triggered electrons are generated to form photocurrent. Afterwards, as the *n*_Ag NP@Au NCs_ further increases, the performance of photocurrent decreases. Reasons can be given as that the resistance of the whole working electrode is also increased resulting in the drop of photocurrent. At last, the incident photon‐to‐electron conversion efficiency (IPCE) measurements are finished using the optimized Ag NP size and *n*_Ag NP@Au NCs_ in Figure 2g. It confirms the stronger photoelectrical property from the optimized Ag NP@Au NCs nanocomposites than plain Au NCs. Thus, building on the above‐mentioned discussion, the increased photocurrent from Ag NP@Au NCs can be obtained on basis of the optimizations.

At last, the practicality of the PEC‐DFM hybrid‐sensing system is further validated through DFM measurements. First, the scattering spectrum from an individual Ag NP@Au NCs nanocomposite is acquired in Figure 2h, using internal light excitation from DFM and selecting a representative Ag NP at random. A summary of recent single‐nanoparticle studies employing DFM is provided in Table S1. Au and Ag nanoparticles are widely employed in DFM systems, with sizes as small as ≈10 nm for both [44]. Given the superior photocatalytic activity of Ag NPs, this study focuses exclusively on silver‐based nanoparticles to maximize PEC signal generation. Considering the Ag NP size‐dependent photocurrent response shown in Figure 2e, a 15.7 nm Ag NP is adopted to enable observation of the scattering spectrum from the Ag NP component in the Ag NP@Au NCs nanocomposite. Three key parameters—*λ*_max_, *I_λ_*_max_ and *Γ*—are extracted for the spectra of each Ag NP@Au NCs nanocomposite, reflecting changes in the dielectric environment surrounding the Ag NP core [22, 23]. When additional external excitation light is applied to the Ag NP@Au NCs nanocomposite, these parameters exhibit measurable shifts. This behavior arises because the Au NCs on the Ag NP surface are photoexcited, generating additional free electrons at the LUMO level, which subsequently transfer to the Ag NP surface and alter the local dielectric constant. As shown in Figure 2i,j, the nanocomposite containing the smallest Ag NP exhibits the largest changes in Δ*λ*_max_, Δ*I_λ_*_max_ and Δ*Γ*, confirming its superior photoelectronic properties. Furthermore, DFM results corroborate the PEC measurements presented in Figure 2, demonstrating that smaller Ag NPs generate higher‐energy hot electrons and yield stronger photocurrent responses. In conclusion, DFM effectively complements the PEC system by enabling a more accurate estimation of photocurrent from Ag NP@Au NCs and providing mechanistic insights into PEC behavior. Together with the optimized nanoparticle size and number determined previously, the integrated PEC‐DFM platform will be employed in subsequent sections for in‐depth investigation of the photocurrent generation mechanism and for glucose sensing applications.

### 2.3 Mechanism Study of Photocurrent Generation from Single Ag NP@Au NCs Nanocomposite

**FIGURE 3:**
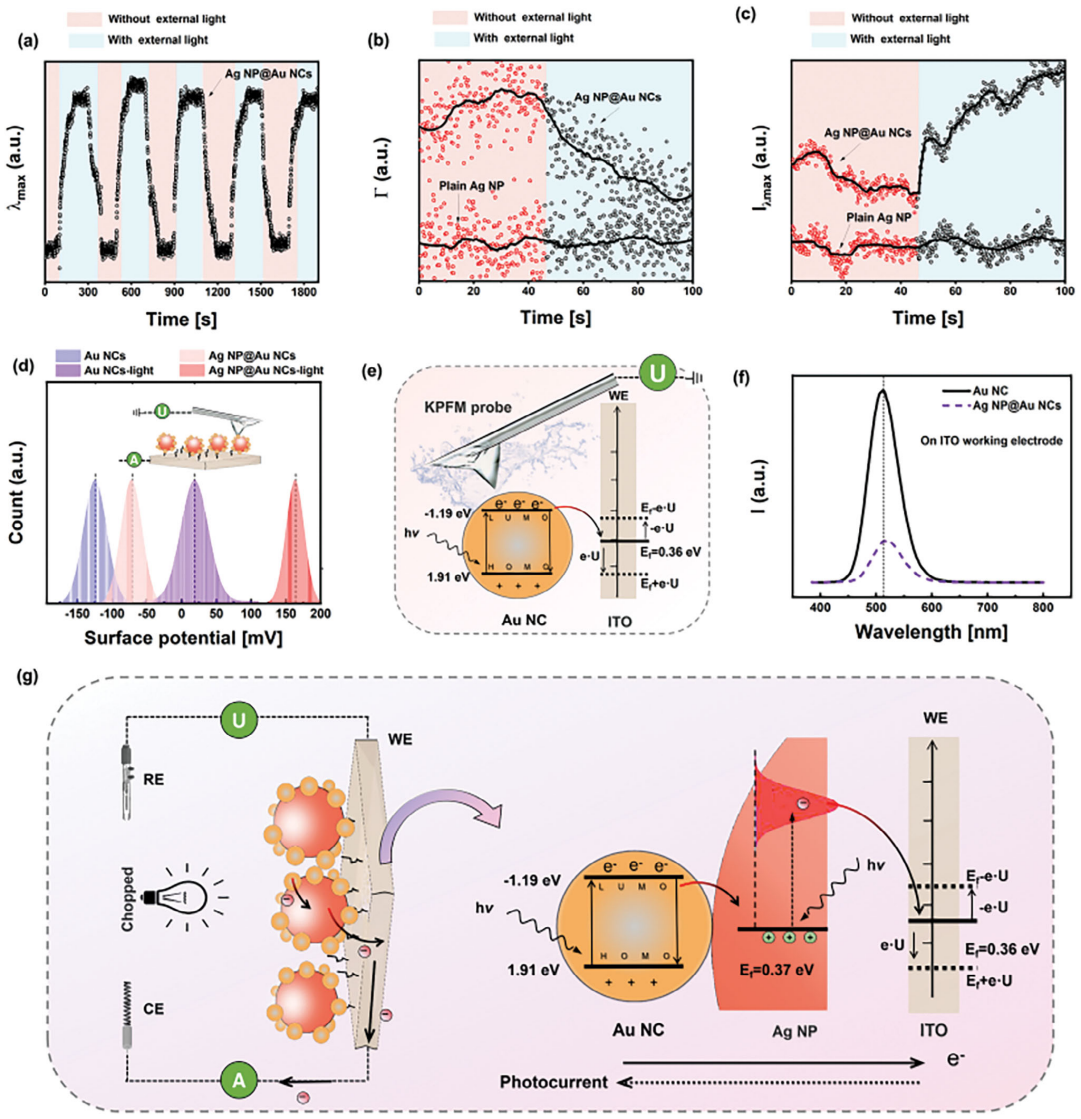
Continuous DFM measurement for Ag NP@Au NCs nanocomposite sample together with three parameters observed including (a) *λ*_max_ (resonance wavelength), (b) *Γ* (width at the half‐maximum of the spectrum) and (c) *I_λ_*_max_ (intensity of the spectrum at *λ*_max_) using internal white light from DFM as base line and extra external light applied as excitation in 0.01 m PBS (pH = 7.4). (d) Surface potential distribution of the working electrode measured by kelvin probe force microscopy (KPFM) after the immobilization of Au NCs and Ag NP@Au NCs. (e) Graphic illustration of photocurrent generation mechanism from Au NCs/ITO electrode in PEC system. (f) The fluorescence measurements from Ag NP@Au NCs/ITO electrode and Au NCs/ITO electrode in the air under an excitation of 375 nm. (g) The graphic illustration of photocurrent generation mechanism from Ag NP@Au NCs on ITO working electrode.

Based on the optimized PEC‐DFM hybrid‐sensing system described above, the photocurrent from Ag NP@Au NCs nanocomposites on an ITO working electrode can be obtained. The corresponding photocurrent generation mechanism is further investigated in this section. The primary objective of this study is to examine the electron transfer behavior at the Ag NP/Au NCs interface within the Ag NP@Au NCs nanocomposite through scattering spectrum analysis using DFM. According to the literature [22, 23], scattering spectra arise from plasmonic metal nanoparticles; thus, electron transfer from Au NCs to Ag NPs can be inferred from changes in parameters derived from the scattering spectrum of Ag NPs in the DFM system. In comparison with previously collected scattering spectra shown in Figure 2i,j, long‐term and continuous measurements of three parameters (*λ*_max_, *I_λ_*_max_, *Γ*) extracted from the scattering spectrum of Ag NP@Au NCs nanocomposites are observed to obtain reliable results presented in Figure 3a–c. The experiments can be summarized as follows: when white light from DFM is used for excitation, continuous outputs (*λ*_max_, *I_λ_*_max_, *Γ*) extracted from the scattering spectrum of Ag NP@Au NCs nanocomposites are considered as baseline values. Subsequently, additional external light is applied and changes in *λ*_max_, *I_λ_*_max_, *Γ* are observed over an extended period.

First, in Figure 3a, the electron transfer inside the nanocomposites can be characterized by the resonance wavelength (λ_res_) change from single noble metallic NP spectrum. The λ_res_ is famous for its dependency on the ligands, shape, material and dielectric constant of the environment near the NP [23]. In our experiment, benefited from the Au NCs as a protection outside, the Ag NP@Au NCs nanocomposite is stable within 100 s using the internal light excitation from DFM as shown in Figure 3a, which means that the ligands, shape and material will not change within this time. Thus, when there is no external light excitation applied, continuous outputs of *λ*_max_ (the *λ*_res_ with peak intensity) from the internal light excitation of DFM system can be used as the baseline to study further response. When external light excitation is applied with regular 150 s on‐off operations (Figure 3a), a clear increase of *λ*_max_ can be observed, and reason of *λ*_res_ redshift can be given as the dielectric constant change can be affected by the increased hot electron amounts around the Ag NP surface. In details, when turning on the external light, more Au NCs around the Ag NP will be excited together with the separation of electron–hole pairs inside the Au NCs. This process can change the dielectric constant of the environment of the Ag NPs surface finally. The effects of dielectric constant on the *λ*_max_ can be explained as the formula (1) as follows, where *ℇ*_m_ represents the dielectric constant of the media, *ω*_p_ is the instinct frequency of metal plasma and *ℇ*_∞_ is the constant [45]. When the value of *ℇ*_m_ increases, the *ω*_p_ will decrease and the *λ*_max_ will increase. As shown in Figure 3a, after many rounds of external light on–off as long as 1800 s, the *λ*_max_ output still keeps stable revealing its reliability of data collection. Finally, the discussion above reveals the reasonable electrons transfer from Au NCs to Ag NP inside the Ag NP@Au NCs nanocomposite during the PEC measurements.

(1) $$\omega_{\mathrm{res}}=\sqrt{\frac{\omega_{p}^{2}}{\varepsilon_{\infty }+2\varepsilon_{m}}}$$

Second, the result from *λ*_max_ above can be supported by the width at the half‐maximum of the spectrum (*Γ*) due to the change of the surface damping from Ag NP according to the literature [46]. The *Γ* can be determined by the damping defined by an ability to reduce the oscillations of hot electrons from Ag NPs due to LSPR. The damping effect can be divided in four parts as bulk damping, inter‐band damping, radiation damping and surface damping, respectively. The electron transfer happening at the interface of Au NCs@Ag NP can only change the surface damping of Ag NP. When the external light excitation is not applied in the first 46 s (Figure 3b), the *Γ* output from Ag NP@Au NCs and plain Ag NP excited by the internal light of DFM system are relative stable, so that they can be used as baseline for further *Γ* observation. When the external light is applied since 46 s, the *Γ* change from the Ag NP inside Ag NP@Au NCs nanocomposite is the further evidence of electron transfer from Au NCs to Ag NP. As a control, the *Γ* change from plain Ag NP is relatively disregarded. These results from DFM confirms the superiority of Ag NP@Au NCs in the PEC system shown in Figure 2e–g. Also, the 100 s continuous collection of Γ verifies the electrons transfer from Au NCs to Ag NP surface, supporting the results above based on *λ*_max_ change.

Third, according to the theory of plasmons as dipole antennas, the intensity *I* can also support the experiment results above based on the relation of *I_λ_*_max_≈ 1/*Γ* ^2^ that *I_λ_*_max_ is directly proportional to 1/*Γ* ^2^ [47]. Comparing with *Γ* output,  the square relation implies the *I_λ_*_max_ output has a better sensitivity than *Γ*. When there is no extra external light applied in the first 46 s, both of Ag NP@Au NCs and plain Ag NP show stable *I_λ_*_max_ output. Thus, the *I_λ_*_max_ from the internal light of DFM system can be used as a baseline for further study of *I_λ_*_max_ output from external light excitation. When the external light is applied, as shown in Figure 3c, the *I_λ_*_max_ output from Ag NP@Au NCs exhibits a clear change than the corresponding output from plain Ag NP. Reasonable explanation can be given as that more Au NCs around the Ag NP will be excited together with the movement of electrons from Au NCs to Ag NP. This can be explained by Mie's theory, and the *I_λmax_* can be written as formula as follows.

(2) $$c_{\mathrm{sca},\max}=\frac{3k^{4}V^{2}}{2\pi }\left|{\left(1+i\cdot \frac{3\varepsilon_{m}}{\varepsilon_{p2}}\right)}^{2}\right|I_{\lambda \max}∼R\left(c_{\mathrm{sca},\max}\right)$$

where *c*_sca, max_ is the effective cross section of Ag NP for light scattering, *k* is the wave vector, *ℇ*_p2_ is the imaginary part of complex dielectric function, *I*_sca,max_ is the real part of *c*_sca,max_. When *ℇ*_m_ increases (namely the *λ*_max_ increase as described above) caused by the electrons transferred from Au NCs to Ag NP surface, the *I_λ_*_max_ will increase agreeing well with the results in Figure 3a and supporting the discussion above based on *λ*_max_ and *Γ*. Also, the continuous DFM measurements also verifies the superior performance of the PEC‐DFM hybrid‐sensing system in observing stable and reliable results from small size catalytic nanomaterial such as Ag NP.

Building on the discussion above, kelvin probe force microscopy (KPFM) is also used to study the electron transfer behavior between Ag NP and Au NCs in the Ag NP@Au NCs nanocomposite. KPFM can reflect surface potential distribution on the surface of working electrode. As shown in Figure 3d, without illumination, the surface potential of Au NCs/ITO electrode is negative because of the MUA ligands. When light is applied, the surface potential of Au NCs/ITO electrode becomes more positive. This can be explained as the light‐triggered electrons at conduction band (CB) from Au NCs above are transferring to the ITO electrode below leaving holes in Au NCs [48]. Thus, based on the DFM results and KPFM results, the photocurrent generation mechanism from Au NCs/ITO electrode in PEC system can be given as a graphic illustration in Figure 3e. In contrast, the surface potential of Ag NP@Au NCs immobilized on ITO working electrode are studied and the amounts of Au NCs are controlled equal with the Au NCs/ITO electrode mentioned above. When there is no illumination, the surface potential of Ag NP@Au NCs/ITO electrode is also negative because of the Au NCs surrounded. The surface potential difference of Au NCs electrode and Ag NP@Au NCs /ITO electrode attributes to the surface ligands of Au NCs and Ag NP. When light is applied, the surface potential of Ag NP@Au NCs /ITO electrode is more positive than the value of Au NC/ITO electrode. Reasonable conclusion can be given as that, on Ag NP@Au NCs/ITO electrode, the Ag NP below can grab the light‐triggered electrons of Au NCs resulting in the more positive values of surface potential than the Au NC/ITO electrode. This verifies the electron transfer from Au NCs to Ag NP in Ag NP@Au NCs nanocomposite under illumination, confirming the conclusions given from the DFM measurements mentioned above. Apart from that, the electron transfer at the Au NCs and Ag NP interface in the nanocomposite can also be studied by the fluorescence measurement of Ag NP@Au NCs/ITO electrode and Au NCs/ITO electrode in the air (Figure 3f). As shown in Figure 3f, while controlling the amounts of Au NCs equal, a clear decreasing of fluorescence intensity can be observed from Ag NP@Au NCs/ITO electrode. The unchanged peak wavelength (513 nm) from Ag NP@Au NCs/ITO electrode comparing with Au NCs/ITO electrode indicates that the fluorescence is from the Au NCs in Ag NP@Au NCs nanocomposites. The reason can be given as follows. Under illumination, the electron–hole pairs generated in Au NCs, and hot electrons are excited leaving holes on Ag NP. These holes of Ag NP can grab the light‐triggered electrons of Au NCs resulting in the limited recombination of electron–hole pairs in Au NCs, thereby observing decreased fluorescence from Ag NP@Au NCs/ITO electrode than Au NCs/ITO electrode. Based on the discussion above, the mechanism of photocurrent generation from Ag NP@Au NCs nanocomposite can be given as the illustration in Figure 3g. The energy levels of Au NCs are calculated (LUMO = ‐1.19 eV, HOMO = 1.91 eV, vs SHE) in section II of Supporting Information using Tauc plot analysis and Mott‐Schottky measurement [49, 50]. Together with the Fermi energy level of Ag NP (0.37 eV versus SHE) [51] and ITO (0.36 eV versus SHE) [52], an electron transfer model of photocurrent generation from Ag NP@Au NCs in PEC system can be given in Figure 3g. Based on the electron transfer model, electrode transfer among Au NC, Ag NP and ITO are exhibited. Based on this, the Ag NP@Au NCs sensor can be further used for glucose detection in the next section.

### 2.4 Ultrasensitive and Reversible Detection of Glucose

Glucose has direct correlations with metabolism, so detecting glucose in wound‐secreted body fluids represents a promising approach for monitoring wound healing. However, the small surface area of wounds and limited volume of exudate result in very low glucose concentrations available for each detection. Thus, an ultrasensitive glucose sensor with ultrahigh sensitivity must be developed. Additionally, the sensor's reversibility is essential to minimize glucose consumption and reduce potential damage to the sensor itself [1]. In this paper, by using the optimized photocurrent from Ag NP@Au NCs, a very faint concentration change of glucose can be detected, enabling ultrasensitive glucose detection. A non‐invasive sensing mechanism based on the molecular‐level competition between glucose and dextran for Concanavalin‐A (Con A) is studied to reduce glucose consumption and overcome the inherent disadvantage of the PEC system employing a redox sensing mechanism, which is the potential harm to the sensor.

**FIGURE 4:**
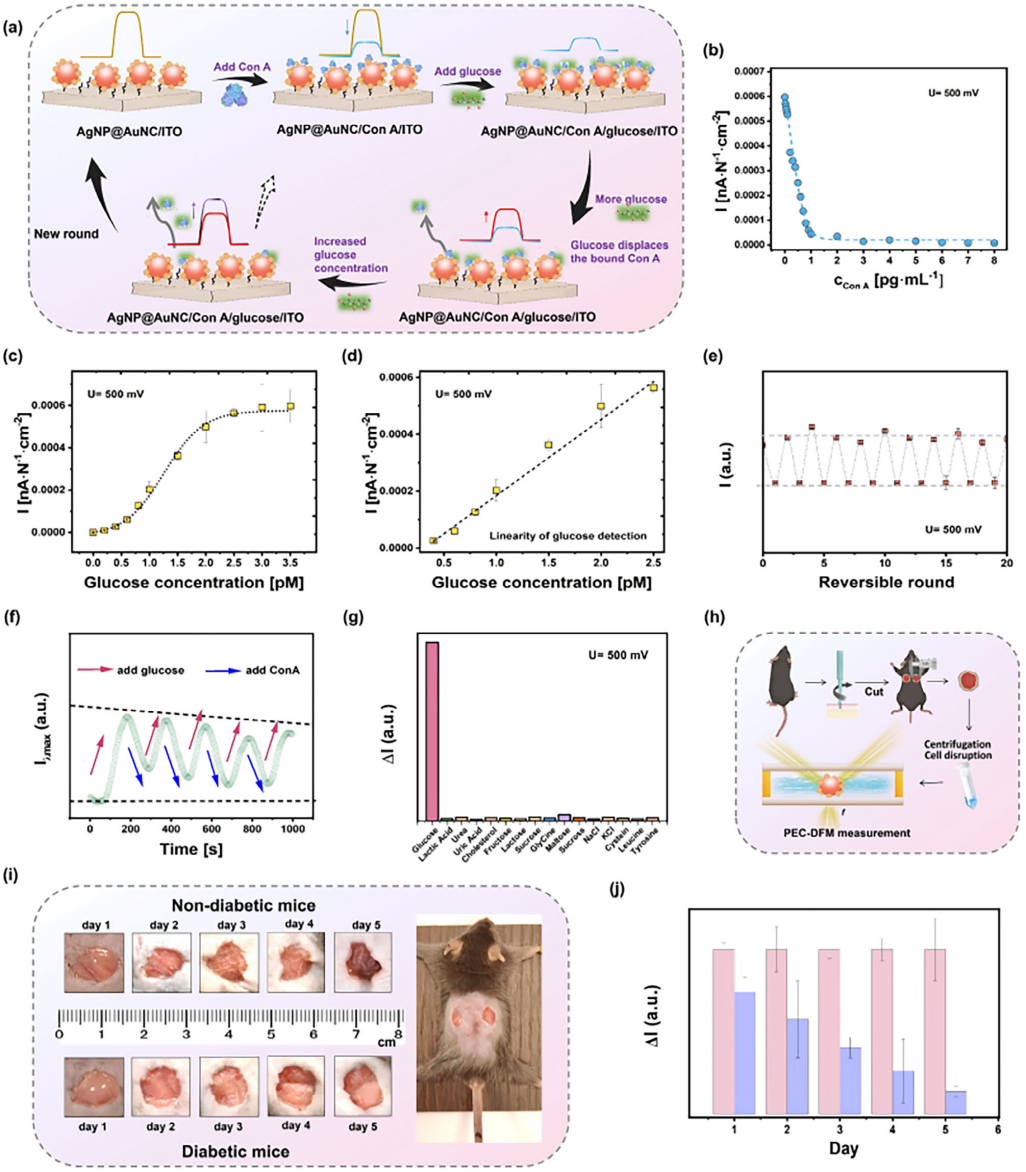
(a) Graphic illustration of the reversible glucose sensing mechanism. (b) The Concanavalin‐A (Con A) absorption on Ag NP@Au NCs/ITO electrode to fabricate the Ag NP@Au NCs/Con A/ITO electrode for further glucose detection. Photocurrent dependence per area from the Con A concentration at *U* = 500 mV (*N* = n_Ag NP@Au NCs_ = 550 708). (c) The glucose detection using Ag NP@Au NCs/Con A/ITO electrode (1 pg · mL^−1^ Con A). Photocurrent response from glucose concentration at *U* = 500 mV in 0.01 m PBS (pH = 7.4) (*N* = *n*_Ag NP@Au NCs_ = 550 708). (d) The corresponding linear range of glucose detection at *U* = 500 mV in 0.01 m PBS (pH = 7.4). (e) The reversible and continuous detection of glucose over 20 consecutive rounds using Ag NP@Au NCs/Con A/ITO electrode using photocurrent from Ag NP@Au NCs at *U* = 500 mV (N = *n*_Ag NP@Au NCs_ = 550 708). (f) The repeated binding and release of Con A on the Ag NP@Au NCs/ITO electrode characterized by DFM using *I_λ_*_max_ from individual Ag NP@Au NCs. (g) Interference study of PEC responses from the Ag NP@Au NCs/Con A/ITO electrode using artificial wound exudate in the presence of a mixture containing common substances (10‐fold than glucose) at *U* = 500 mV. (h) A graphic illustration of the collection of wound tissue fluid from live mice for PEC measurements within the PEC‐DFM hybrid‐sensing system. (i) Representative photo of the wound from living diabetic and non‐diabetic mice in 5 d. (j) The PEC response from the wound tissue extracted from diabetic and non‐diabetic mice over a 5‐day period at *U* = 500 mV (*N* = *n*_Ag NP@Au NCs_ = 550 708).

The graphic illustration of the sensing mechanism is presented in Figure 4a. Overall, Con A is one of the lectins that can reversibly bind to carbohydrates such as glucose and dextran [6, 53]. The binding interaction depends on the structure of carbohydrates (such as α‐D‐glucose), environmental factors (such as pH and metallic ions of Ca^2+^/Mn^2+^), and Concanavalin‐A state (such as polymerization). A good binding affinity can be observed when Con A is immobilized on the surface of an electrochemical sensor as a sensing probe in our previous work [6]. Thus, as the surface of Ag NP@Au NCs is modified with dextran, Con A can be absorbed on the Ag NP@Au NCs/ITO electrode to form the Ag NP@Au NCs/Con A/ITO electrode for further glucose detection. The sensing mechanism can be seen from Figure 4a that as the concentration of Con A increases, the affinity between dextran and Con A (*F*_Dextran‐Concanavalin‐A_) keeps increasing and then reaches a stable value to obtain the Ag NP@Au NCs/Con A/ITO electrode for further glucose detection. As the concentration of glucose increases, more glucose can bind to the Ag NP@Au NCs/Concanavalin‐A/ITO electrode, and the affinity between glucose and Con A (*F*_Glucose‐Concanavalin‐A_) keeps increasing. When *F*_Glucose‐Concanavalin‐A_ is larger than *F*_Dextran‐Concanavalin‐A_, glucose can take away Con A from the working electrode surface. The Ag NP@Au NCs/ITO electrode can be used to start a new round of detection, realizing a reversible method for detecting glucose.

The glucose sensing results in the PEC system based on the photocurrent from Ag NP@Au NCs can be seen in Figure 4b–d. Initially, the Con A concentration in the PEC sensor is optimized in Figure 4b. As the concentration of Con A rises steadily, more Con A macromolecules are absorbed on the Ag NP@Au NCs/ITO electrode because of *F*_Dextran‐Concanavalin‐A_ increases. The photocurrent decreases dramatically due to an increase in the resistance of the whole Ag NP@Au NCs/Con‐A/ITO electrode, which can be verified by the larger semicircle diameter of electrochemical impedance spectroscopy (EIS) measurements and the smaller current of cyclic voltammetry (CV) results in Figures S10 and S11. When the Con A concentration reaches 1 pg · mL^−1^, the photocurrent keeps stable and the Con A macromolecules absorbed on the electrode have reached saturation. Thus, this concentration of Con A is selected to fabricate the Ag NP@Au NCs/Con A/ITO electrode for further glucose detection. The glucose sensing results can be seen in Figure 4c. At first, as the concentration of glucose gradually increases, *F*_Glucose‐Concanavalin‐A_ becomes larger and consequently, when *F*_Glucose‐Concanavalin‐A_ is larger than the *F*_Dextran‐Concanavalin‐A_, photocurrent increases accordingly, indicating that resistance of the whole electrode decreases and that Con A is taken away by glucose. This can be verified by the smaller semicircle diameter of EIS and larger current from CV results in Figures S10 and S11. Afterwards, as *F*_Glucose‐Concanavalin‐A_ increases, the rate of photocurrent increase accelerates. It should be noticed that the decreased photocurrent before from Con A can also be explained as the block of light excitation by Con A macromolecules absorbed on the electrode surface (Figure S12). Finally, the photocurrent reaches its maximum value and remains stable revealing that most of the Con A macromolecules are removed from the electrode. The linear range of glucose detection is from 0.4 to 2.5 pm as shown in Figure 4d with a sensitivity of 2.67 nA · N^−1^ · cm^−2^. Based on this, the detection limit (LOD = 3×standard deviation/sensitivity) can be calculated as 0.49 pm.

Moreover, the reversibility of the glucose sensor is further studied in the PEC system as shown in Figure 4e. Using 1 pg · mL^−1^ Con A to build the Ag NP@Au NCs/ Con A/ITO electrode, glucose can be continuously detected with reversible additions of 0.4 and 2.5 pm of glucose. Eventually, 20 reversible detection cycles can be observed under the PEC system, which is acceptable compared with recently reported reusable sensors in Table S2. Additionally, the reversible sensing mechanism can be further verified by EIS measurement when 10 rounds reversible glucose in Figure S10. Reversible semicircle diameter changes suggest the impedance changes and the release/binding of Con A on the electrode. Also, CV measurements were performed in Figure S11 for 25 reversible detection cycles, and the corresponding reversible current changes further support the EIS results. Moreover, during the first 2 to 20 rounds, the current remains relatively stable. After 20 rounds, the current response deteriorates. Eventually, during the 25th round, the current decreases dramatically, suggesting that Con A cannot be completely removed and that the sensor described in this paper can be reused only a limited number of times. In addition, UV‐vis spectra for Ag NP@Au NCs/Con A/ITO electrode and the Ag NP@Au NCs/Con A/glucose/ITO electrode are used to characterize the Con A modification and glucose detection. The related results support the EIS and CV measurements. At last, the reversible detection can be verified by studying individual Ag NP@Au NCs using a DFM system to obtain continuous scattering light intensity (*I_λ_*_max_) output over 1000 s. Based on the changes in *I_λ_*_max_, the repeated binding and release of Con A on the Ag NP@Au NCs/ITO can be seen in Figure 4f. This confirms the reversible measurement in the PEC system shown in Figure 4e, and it verifies that the competition‐based non‐invasive sensing mechanism is effective.

In the meantime, the representative glucose sensors published in the last 3 years are summarized in Table 1, including both optical and electrical types. The former includes fluorescence assays, colorimetric methods, and SPR methods. The latter consists of electrochemical (EC) methods and PEC methods. For all of them, enzymatic sensors and optical usually show very low LOD, such as 1 fm from Chen et al. using the SPR method [7] and 0.13 µm using enzymes [54]. Additionally, both EC and PEC methods demonstrate excellent performance. Nevertheless, their reversibility should be further improved. In this paper, an ultrasensitive and reversible glucose sensor is developed with good performance that has advantages over recent years' research. Thus, based on this development, further studies will use this sensor to monitor wounds in living mice. To achieve this goal, the interference of the Ag NP@Au NCs nanocomposite‐based PEC sensor is studied by measuring a mixture of glucose and other common substances found in wound exudate from body fluids; a good selectivity for glucose can be successfully observed in Figure 4g. Meanwhile, the stability of the sensor was also investigated. As shown in Figure S13, the photocurrent remained stable even under continuous illumination for 1000 s. Based on these findings, wound exudates collected from living mice with varied blood glucose levels are detected as shown in Figure 4h–j. Two groups of mice‐diabetic and non‐diabetic are studied over a period of 5 days (Figure 4i). The number of biological replicates (*n* = 5) per group, and the *p*‐value is 0.0012, indicating a statistically significant difference in the PEC response between the two groups. It can be seen that the healing speed of diabetic mice is obviously slower with a relatively constant wound area compared to non‐diabetic ones due to their high blood sugar levels which are usually within the range of mm concentrations [55, 56]. It should be noticed that the rather narrow linear range established in this work is not suitable for portable sensor in rapid, on‐site detection, and this paper is focused on laboratory‐based sensing system in which appropriate dilution can be applied to bring the analyte into the effective linear range. Figure 4h illustrates extracting wound tissues from living mice. The extracted tissues are processed and then measured within the PEC system shown in Figure 4j. For diabetic mice, the PEC output remains stable during these five days while revealing consistently higher glucose levels; whereas for non‐diabetic ones, photocurrent decreases as wound areas become smaller with lower glucose concentrations. Thus, these results indicate that the home‐built glucose sensor presented in this paper can be used for wound‐related detection involving small volumes of body fluid at low concentrations of glucose.

**TABLE 1:** The representative literature of the glucose sensor in recent three years.

Method	Transducer	Nanomaterial	LOD	Reversible	Enzymatic	Refs.
Electrical	PEC	Ag NP@Au NCs	0.49 pm	Yes	No	This work
		Ni‐MOF/α‐Fe_2_O_3_	0.9 µm	No	No	[54]
		Ni(OH)_2_/Fe_2_O_3_	2.2 µm	No	No	[57]
		NiO/Mo‐POM	0.33 nm	No	No	[11]
		CuO@TiO_2_ HNT	0.7 µm	No	No	[58]
		GOX/CuO@BiVO_4_	0.13 µm	No	YES	[54]
		Sn_3_O_4_/CdS	0.067 µm	No	No	[59]
	EC	Ga‐PEDOT‐Pt	10 µm	No	YES	[60]
		Nf/GOx‐ZnO/Ag	4.6 µm	No	YES	[61]
		NF@CoO	0.55 µm	No	No	[62]
Optical	Fluorescence	FP@Bi‐BDC‐NH_2_@Au	4.2 µm	No	No	[9]
	Colorimetry	BC‐CMC‐Ch	0.1 µm	No	YES	[8]
	SPR	TFBG/GO/PBA	1 fm	No	No	[7]

## 3 Conclusions

In summary, we have developed a groundbreaking PEC‐DFM hybrid‐sensing system that enables simultaneous single photocurrent measurement by PEC system and single‐nanoparticle monitoring by DFM system. This platform guided the optimization of Ag NP@Au NCs nanocomposites, yielding a 193‐fold SNR enhancement and allowing the direct observation of electron transfer from Au NCs to Ag NPs. Leveraging these insights, we engineered a non‐invasive, competition‐based sensing mechanism, achieving ultrasensitive (LOD = 0.49 pm) and fully reversible (20 cycles) glucose detection. Successfully validated with real wound exudate, this work not only presents a superior biosensor for wound management but also establishes a versatile paradigm for combined studies in PEC and DFM science.

## 4 Experiments and Materials

### 4.1 The Synthesis of Au NCs

Firstly, 45 mL of deionized water was added to a flask, followed by the sequential addition of 0.5 mL of sodium hydroxide (NaOH) (1 m, CAS:1310‐73‐2) and 1 mL of tetrakis(hydroxymethyl)phosphonium chloride (THPC) (CAS: 124‐64‐1) solution (prepared by dissolving 12 µL of 80% THPC in 1 mL of Milli‐Q water). After stirring for 5 min, 1.5 mL of a 1.0 wt% HAuCl_4_ (CAS: 16961‐25‐4) solution was introduced into the mixture. Following an additional minute of continuous stirring, the solution gradually turned brown. Subsequently, in a separate step, 5 mL of the reaction mixture, together with 1 mL of sodium tetraborate (50 mm, CAS:1330‐43‐4) and 1 mL of MUA (5 mm, CAS:71310‐21‐9), were sequentially added to 3 mL of MilliQ water. The resulting solution was allowed to react at room temperature for 72 h in the dark to yield a colorless Au NC solution, which was then purified by repeated ultrafiltration and centrifugation (10000 rcf, 10 min). The resulting Au NCs were characterized by UV‐vis spectroscopy and DLS. Finally, the Au NCs were collected and further purified by repeated centrifugal filtration (8000 rcf, 20 min) [63].

### 4.2 The Synthesis of Different Sizes of Ag NP

Firstly, the Ag seed was synthesized as follows: 100 mL of Milli‐Q water containing sodium citrate (SC) (5 mm, CAS:6132‐04‐3) and tannic acid (TA) (0.1 mm, CAS:1401‐55‐4) was added to a three‐necked round‐bottom flask. The flask was heated using a heating jacket under magnetic stirring for 15 min, followed by the addition of 1 mL of AgNO_3_ (25 mm, CAS: 7761‐88‐8) after boiling. The solution immediately turned bright yellow and was allowed to react completely for 24 h at room temperature in the dark. The resulting Ag NPs were purified by repeated centrifugation (5000 rcf, 7 min). Subsequently, the synthesized Ag seed and 16.5 mL of Milli‐Q water were transferred to another three‐necked round‐bottom flask and heated to 90°C in a water bath. While maintaining this temperature, 500 µL of SC (25 mm), 1.5 mL of TA (2.5 mm), and 1 mL of AgNO_3_ (25 mm) were sequentially added to the solution. Repeating this growth procedure with the resulting solution enabled the synthesis of Ag NPs with tunable sizes. After that, approximately 0.19 nm dextran‐thiol (≈6 kDa; purchased from Xian Qiyue Biology) was conjugated to the Ag NPs via incubation at 60°C for 3 h under dark conditions. The obtained Ag NPs were characterized by UV–vis spectroscopy and DLS. Finally, the nanoparticles were purified through repeated centrifugation (7000 rcf, 7 min) [35].

### 4.3 The Synthesis of Ag NP@Au NCs Nanocomposite

A volume of 12 mL of Ag NPs (0.33 mm) solution was transferred into a 20 mL glass vial, followed by the addition of 3 mL of poly(allylamine hydrochloride) (PAH) (1% wt, CAS: 71550‐12‐4) under vigorous stirring for 15 min. The excess PAH was subsequently removed via centrifugation (5000 rcf, 7 min) [36]. Next, MUA‐Au NCs (100 µL, 0.55 µM) were mixed with the modified Ag NPs (ranging from 0 to 8 µL) and incubated in the dark for 30 min to facilitate conjugation. The resulting mixture was then sonicated for 5 min at room temperature and purified through repeated centrifugal filtration to eliminate unbound Au NCs and residual PAH. Finally, the resulting Ag NP@Au NCs nanocomposite was stored at 4°C for subsequent use.

### 4.4 The Fabrication of Working Electrode

The ITO glass (1.5 cm × 3 cm) was sequentially sonicated in Triton X‐100 (20% aqueous solution), Milli‐Q water, and ethanol (CAS: 64‐17‐5) for 7 min each to ensure thorough cleaning. Subsequently, the electrodes were dried under a stream of N_2_ and treated with a mixture of NH_4_OH, H_2_O_2_, and H_2_O (1:1:5) at 80°C for 30 min to introduce hydroxyl (─OH) groups onto the ITO surface [42]. After rinsing with Milli‐Q water and drying with N_2_ flow, the cleaned electrodes were immersed in a 3‐MPTMS (≈3%, CAS: 4420‐74‐0) solution containing silane coupling agents for 12 h in the dark, followed by N_2_ drying to facilitate the formation of thiol (─SH) functional groups via covalent bonding between the silane moiety of 3‐MPTMS and the surface ─OH groups. Finally, following another rinse with Milli‐Q water and drying under N_2_, the activated ITO electrodes were immersed in Ag NP@Au NCs solutions for a 30 min incubation period to enable nanoparticle immobilization. Unbound Ag NP@Au NCs were removed by gentle rinsing with Milli‐Q water, and the resulting Ag NP@Au NCs/ITO electrode was dried under a nitrogen stream for subsequent use [64]. The working electrode fabricated can be used for further sensing experiments. ‐

### 4.5 Statistical Analysis

All photocurrent data are presented as mean values and error bars obtained from 5 separate measurements.

## Ethics Statement

Ethical approval for the animal experiments has been obtained from the Ethics Committee of Yanshan University (Ethics Review No.: 202303‐015), and all participants provided informed consent.

## Conflicts of Interest

The authors declare no conflicts of interest.

## Supporting information




**Supporting File**: advs74885‐sup‐0001‐SuppMat.docx.

## Data Availability

The data that support the findings of this study are available from the corresponding author upon reasonable request.
