# Supplementary material for: PEC‐DFM Hybrid‐Sensor Based on Nanoparticle‐Resolved Estimation for Sensitive and Reversible Glucose Monitoring
Source: Adv Sci (Weinh). 2026 Mar 24;13(31):e23818. doi: 10.1002/advs.202523818 (PMC13587973; doi:10.1002/advs.202523818)
Supplement: Supplementary file 1 [file ADVS-13-e23818-s001.docx]

**PEC-DFM Hybrid-Sensor based on Nanoparticle-Resolved Estimation for Sensitive and Reversible Glucose Monitoring**

Zihao Zhang^1^, Junzheng Liu^1^, Qinggong Wu^1^, Yuan Zeng^3^, Gang Xiao^2^, Zhao Yue^2,*^, Zhigang Zang^1,4,*^, Shuang Zhao^1,*^

^1^ Key Laboratory for Special Fiber and Fiber Sensor of Hebei Province, School of Information Science and Engineering, Yanshan University, 066004 Qinhuangdao, PR China

^2^ Department of Microelectronics, Nankai University, 30071 Tianjin, PR China

^3^ College of New Materials and New Energies, Shenzhen Technology University, 518118 Shenzhen, PR China

^4^ College of Optoelectronic Engineering, Chongqing University, 400044 Chongqing, P. R. China

^*^ Corresponding authors: zangzg@cqu.edu.cn

**Supporting Information**

I) Overview of the PEC-DFM hybrid-system

II)	Energy level calculation

III)	Additional characterizations

IV)	References

I) **Overview of the PEC-DFM hybrid-sensing system**

The core advantage of dark-field microscopy (DFM) lies in its highly sensitive scattering-based imaging of nanoscale objects. Owing to the distinctive "dark background, bright sample" contrast mechanism, DFM achieves a high signal-to-noise ratio and spatial resolution down to tens of nanometers. The fundamental principle of photoelectrochemical (PEC) systems centers on the generation and regulation of photogenerated charge carriers: under illumination, semiconductor photoelectrodes produce electron-hole pairs that drive redox reactions via interfacial charge transfer. The synergistic integration of DFM and PEC systems represents a powerful convergence of "visual observation" and "controllable reaction." Specifically, in this paper, the PEC system provides a tunable photo-electro-chemical environment that enables dynamic modulation for DFM observation, while DFM delivers monitoring of nanoparticle behavior during the PEC process. In this paper, the integrated system of PEC and DFM is capable of output the number (n_AgNP@AuNCs_) from the DFM module and photocurrent (I_AgNP@AuNCs_) from the PEC module. Eventually, the photocurrent from a single AgNP@AuNCs nanocomposite can be estimated as I_single_=I_AgNP@AuNCs_/n_AgNP@AuNCs_.

**Table S1** Comparisons of the nanomaterial and size from the metal nanoparticles in DFM system in recent three years.

| **Nanomaterial** | **Size** | **System** | **Application** | **Ref.** |
| --- | --- | --- | --- | --- |
| AgNP | 15.7 nm | PEC-DFM | Detection of glucose | **This work** |
| AgNP | 80 nm | DFM | Detection of Human Papillomavirus | ^[1]^ |
| AgNP | 40 nm | DFM | Galvanic exchange process visualization | ^[2]^ |
| AgNP | ~45 nm | DFM | Investigating a reaction that is inconspicuously weak, slow, or coexists | ^[3]^ |
| AgNP | ~35 nm | DFM | Photoconversion and photocatalysis study | ^[4]^ |
| AgNP | ~30 nm | DFM | Photocatalysts and nanosensors study | ^[5]^ |
| AgNP | 11 nm | DFM | Nano-toxicological study | ^[6]^ |
| AuNP | 100 nm | DFM | Quantification of Virus-Like Particles | ^[7]^ |
| AgNP | 76 nm | DFM | Detection of Aldehyde Compounds | ^[8]^ |
| AuNP | 40 nm | DFM | Detection of C-Reactive Protein | ^[9]^ |
| AuNR | 88.8 nm | DFM | Improving the Detection Limit | ^[10]^ |
| AuNP | 50 nm | DFM | Glucose Detection | ^[11]^ |
| AuNP | 28.21 nm | DFM | Pathogen Detection | ^[12]^ |
| AuNS | 20 nm | DFM | DFM Imaging Scatterometry | ^[13]^ |
| AuNR | 30 nm | DFM | Detection of MicroRNA-21 | ^[14]^ |
| AuNP | 7-9 nm | DFM | Uptake of Gold Nanoparticles | ^[15]^ |

Notes: Silver nanoparticle (Ag NP), gold nanoparticle (Au NP), gold nanospheres (AuNS)，gold nanorods (AuNR).

**Table S2** Representative reversible sensors published in recent years.

| **Transducer** | **Nanomaterial** | **Analyte** | **Small molecule** | **Cycles** | **Ref.** |
| --- | --- | --- | --- | --- | --- |
| Photoelectrochemistry | AgNP@AuNCs | Glucose | Biological affinity | 20 | **This work** |
|  | CdTe-MWNTs | SO_2_ | Redox reaction | 10 | ^[16]^ |
|  | Bi₂S | TMB | Redox reaction | 4 | ^[17]^ |
| Electrochemistry | Lys | CO₂ | Chemical bond | 10 | ^[18]^ |
|  | Fe-CuO | H_2_S | Redox reaction | 18 | ^[19]^ |
|  | 2D MPc-O_8_-Cu MOF | CO | Redox reaction | 14 | ^[20]^ |
| Optical | QG-1 | GSH | Enzymatic Reaction | 2 | ^[21]^ |
|  | BTPP | Cu^2+^ | Chemical bond | 10 | ^[22]^ |
|  | UCNP@mSiO_2_ | Glucose | Biological affinity | 8 | ^[23]^ |
|  | UCNPs | Sulfitet | Chemical bond | 10 | ^[24]^ |
|  | BSA- Trz | Pb^2+^ | Chemical bond | 6 | ^[25]^ |
| QCM | Ni^2+^ | His-tagged | Chemical bond | 4 | ^[26]^ |

Notes: Lysine (Lys), metal–organic frameworks (MOFs), a ratiometric fluorescent probe (QG-1), 2-(benzo[d]thiazol-2-yl)-6-(7,8,13,14-tetrahydrodibenzo phenanthridin-5-yl) phenol (BTPP), upconversion nanoparticles (UCNPs), benzenesulfonic acid (BSA), 1H-1,2,4-triazole-3,5-diamine (Trz), 3,3′,5,5′-tetramethylbenzidine (TMB), glutathione (GSH).

**Figure S1** The illustration of using (a) plain Ag NP and (b) AgNP(PAH) to fabricate (c) AgNP@AuNCs nanocomposite. (d) Sketch of the AgNP@AuNCs/ITO working electrode fabrication. The side view of the AgNP@AuNCs/ITO working electrode exhibits the distance (d) between different AgNP@AuNCs and size (2×r_AgNP@AuNCs_) when the nanomaterials are approximated as spherical shapes and AgNP@AuNCs on the chip are evenly distributed at equal distance intervals under ideal conditions. (e) The corresponding top view of the AgNP@AuNCs/ITO working electrode. (f) The experimental images from AgNP@AuNCs are captured using DFM and the bright spots in the images represent individual AgNP@AuNCs. The mean value of distance intervals (d_mean-DFM_) is between different AgNP@AuNCs measured from DFM.

The accuracy of the I_single_ is controlled through four combined approaches.

(1) First, image recognition technology is introduced including four basic steps in the algorithm as image preprocessing, region segmentation, feature extraction/screening, counting and output.
(2) Second, for a silver nanosphere with a well-defined size, the parameters (*λ_max_*, *I_λmax_*, *Γ*) from scattering spectrum is known and defined as standard values. In the measurements, the scattering spectra of each nanoparticle will be collected, thereby obtaining the parameters (*λ_max_*, *I_λmax_*, *Γ*) of each individual AgNP@AuNCs nanocomposites. These values will be compared with the standard values, thereby eliminating the aggregated nanoparticles and correcting the n_AgNP@AuNCs_ value.
(3) Third, the AgNP@AuNCs/ITO electrodes are further characterized by ICP-MS to obtain the mass of Ag and Au on the working electrode, and then the n_AgNP@AuNCs_ can be characterized as follow. Considering the AgNP as a standard spherical shape^[27]^, together with the size characterized by TEM (d_c_=15.7 nm), the mass of single AgNP is m_AgNP_=V_AgNP_×ρ_Ag=_(4/3)×π×(d_c_/2)^3^×ρ_Ag=_2.1·10^-17^ g, using the bulk density of silver of ρ_Ag_ = 10.49 g/cm^3^. The molar mass of one AgNP unit is M_Ag_=107.87 g/mol. Based on the Avogadro's number N_A_ = 6.02·10^23^ mol^-1^ the number of Ag per Ag NP is N_Ag/AgNP_ = (m_AgNP_/M_Ag_)·N_A_ ≈ 117200. The AgNP@AuNCs/ITO working electrode will be immersed in V_sol_ (mL) aqua regia for 24 h to digest the AgNPs. After taking ITO out from the aqua regia solution, the Ag mass concentration (C_Ag_) in the solution can be measured with ICP-MS as C_Ag_ considering the density of water of 1 g/cm^3^. The total mass of Ag on the electrode is m_AgNP/chip_ = C_Ag_·V_sol_. The number of Ag NPs per electrode is N_AgNP/chip_ = m_AgNP/chip_/(N_Ag/AgNP_·M_Ag_/N_A_)=550708 and based on this, the distance between different AgNP@AuNCs nanocomposites can be estimated according to the illustration shows in Figure S1. Firstly, the synthesized AgNP@AuNC (Figrue S1a-S1c) can be used to fabricate working electrode. After AgNP@AuNCs are immobilized on ITO electrodes (Figrue S1d and S1e), the distance (d) between individual AgNP@AuNCs nanocomposites can be seen in Figure S1d and S1e, that the AgNP@AuNCs nanocomposites are considered evenly distributed at equal distance intervals under ideal conditions. For a single AgNP@AuNCs, the occupied area is S_AgNP_=[2×(d/2)+2×r_AgNP@AuNCs_]^2^ as shown in Figure S1e. When the working electrode area (S_working electrode_) is considered as 1 cm^2^, so S_AgNP_=S_working electrode_/N_AgNP/chip_ and the distance between different AgNP@AuNCs nanocomposites is calculated as d=13.4596 µm. Additionally, based on the experimental images from AgNP@AuNCs captured using the CCD camera of DFM, the mean value of distance intervals (d_mean-DFM_) between different AgNP@AuNCs measured can be measured as 14.24±3.6 µm. The closed value between d and d_mean-DFM_ discussed above reveals that the n_AgNP_ calculated in this paper is quite accurate. The distance value (d_mean-DFM_) is much greater than the size of AgNPs, verifying that the aggregation between AgNP@AuNCs nanocomposites can be ignored. Thus, the photocurrent output from single AgNP@AuNCs (I_single_) in this paper can be used.

**Figure S2** (a) Workflow diagram for single-nanoparticle spectroscopy in the PEC-DFM hybrid-sensing system. (b) Identification of nanoparticles on the ITO working electrode using image recognition. The inserted images depict the disappearance of the scattering light spot due to nanoparticle damage. (c) The related computer software of the PEC-DFM hybrid-sensing system. (d) An example of the time-dependent *λ_max_* output obtained using a damaged nanoparticle.

In the PEC-DFM hybrid-sensing system, a final photocurrent is generated (I=I_Ag NP@Au NCs_/n_Ag NP@Au NCs_). It shoud be noticed that in order to facilitate the comparison between the two materials of AuNCs and AgNP@AuNCs (such as in Figures 2e and 2f), the photocurrent of AuNCs in Figure 2b is calculated as I/N (N=n_Ag NP_). The n_Ag NP@Au NCs_ in the formula is essential and is derived from the DFM module, whose workflow diagram is shown in Figure S2a. Based on this, nanoparticles self-assembled on the ITO working electrode are identified using image recognition (Figure S2b), enabling exclusion of damaged Ag nanoparticles and thereby improving the accuracy of n_Ag NP_ quantification. The computer software is shown in Figure S2c. Damaged Ag nanoparticles are identified by both the disappearance of the scattering light spot (see inserted image in Figure S2b) and the λ_max_ drift (drift=Δλ/λ_max-final_=(λ_max-initial_-λ_max-final_)/λ_max-final_) (Figure S2d).

An example of the λ_max_ drift calculation for a damaged nanoparticle is shown in Figure 2d, where a substantial drift (96.8%) leads to the exclusion of this nanoparticle from the n_Ag NP@Au NCs_ population.

**Figure S3** Open circuit potential (OCP) between working electrode and Pt reference electrode in the PBS (pH=7.4) at 24℃.

For the PEC cell, 0.7 mm ITO glass is used to build working electrode (WE), and 0.2 mm Pt wire is used as reference electrode (RE) and counter electrode (CE). The RE can provide a stable potential when the redox reaction reaches a dynamic equilibrium^[28]^. The electrode potential corresponding to this state is the open-circuit potential (open-circuit potential, OCP)^[29]^. If the OCP can remain stable for a long time, the feasibility of RE can be reasonably inferred. The OCP test in Figure S3 using Pt as the RE (24℃, PBS, pH = 7.4) remain basically stable over 400 s. Therefore, it proves that the Pt of RE in this paper is reasonable.

The signal-to-noise ratio (SNR) of a PEC system refers to the ratio of the effective intensity of the target PEC response signal (such as photocurrent) detected by the system to the background signal (such as dark current) of the system. The calculation formula is SNR=photocurrent/darkcurrent. This parameter focuses on the "light-electrode" conversion and signal acquisition in PEC processes. Its value is mainly determined by the performance of the photoelectrode in this paper. The SNR of Au NC and Ag NP@Au NCs photoelectrodes are calculated as follows.

For plain Au NC, its SNR can be calculated as:

SNR_AuNC_=photocurrent/darkcurrent≈9.9E-05

For Ag NP@Au NCs, its SNR can be calculated as:

SNR_Au NC@Ag NP_=photocurrent/darkcurrent≈1.91E-2

Thus, the SNR improvement can be calculated by SNR_Au NC@Ag NP_/SNR_AuNC_≈193

**Figure S4** The signal-to-noise ratio (SNR) of the PEC system from Au NCs and AgNP@AuNCs

II) **Energy level calculations**

As shown in Figure S2, the electronic band structure of MUA-AuNCs was characterized by combining Tauc plot analysis with Mott-Schottky measurements. The Tauc plot, derived from the UV-visible absorption spectrum, yielded an optical band gap (Eg) of 3.1 eV. Furthermore, analysis of the Mott-Schottky curve provided the energy positions of the valence band (VB) and conduction band (CB), which were determined to be 1.91 eV and -1.19 eV (vs. SHE), respectively.

**Figure S5** (a) UV-vis absorption spectroscopy result, (b) Mott-Schottky measurements and (c) Tauc plot results from MUA-AuNCs in solution.

**III) Additional characterizations**

**Figure S6** Atomic force microscope (AFM) measurements from Au NCs immobilized on the ITO working electrode.

The size difference between TEM and AFM is attributed to the measurement mechanisms of TEM and AFM. TEM characterizes the inorganic gold part of the gold nanoclusters, excluding the surface ligands. AFM operates by detecting the van der Waals force between the probe and the gold nanoclusters, including not only the gold part but also the surface-capping ligands. Thus, considering the thickness of the MUA ligands, it is reasonable that the AFM results of gold nanoclusters (3.65±0.3 nm) appears larger than the TEM one (2.1±0.2 nm) ^[30]^.

Pure Ag NPs are susceptible to degradation in PEC systems due to electrons transfer, leading to structural collapse. In contrast, the Au NCs modified on the Ag NP act as robust physical barrier, and effectively isolate the Ag NPs from the electrolyte in PEC measurements and inhibits the oxidation and corrosion.

**Figure S7** The particle size distribution curve obtained from the AFM images of (a) AgNP/ITO as 16.6±1.58 nm and (b) Ag NP@Au NCs/ITO as 23.9±1.53 nm.

**Figure S8** Dynamic light scattering (DLS) results from (a) Au NCs, (b) AgNPs (d_c_=15.7 nm) and (c) AgNP@AuNCs.

**Figure S9** Dynamic light scattering (DLS) results from (a) 27.3±2.3 nm, (b) 48.5±6.3 nm, (c) 80.5±5.1 nm, (d) 100.3±7.2 nm AgNP.

**Figure S10** Electrochemical impedance spectroscopy (EIS) measurements of different electrodes during 10-cycle reversible glucose detection in the dark after 2.5 pM glucose is added and detected.

Electrochemical impedance spectroscopy (EIS) is an important characterization, capable of characterizing charge transfer properties^[31]^. The high-frequency region of EIS typically exhibits a semicircular feature, with the diameter corresponding to the charge transfer resistance of the redox probe at the electrolyte interface. The smaller the semicircle diameter, the faster the separation rate of electron-hole pairs and the smaller impedance of the whole electrode^[32]^.

To further understand the competitive mechanism of dextran and glucose against Con A, the electronic transfer at the sensing interface was studied through electrochemical impedance (Figure S10). It can be observed that when Coceptible to degradation in PEC systems due to electrons transfer, leading to structural collapse. In contrast, the n A is added, the semicircle arc becomes larger, indicating an increase in electrode interface impedance, thereby affecting the reduction of the photocurrent signal; when glucose is added, the semicircle arc becomes smaller, indicating that glucose competes to remove Con A, resulting in a decrease in electrode interface impedance. After repeating the competition process 10 times, the semicircle arc is almost the same as before, proving the stability of the competitive mechanism. This result supports the mechanism mentioned in Figure 4a.

**Figure S11** Cyclic voltammetry (CV) measurements of different electrodes during 25-cycle reversible glucose detection in the dark after 2.5 pM glucose is added and detected.

Cyclic voltammetry (CV) is a widely used electroanalytical technique that investigates the redox behavior of electroactive species at the electrode–electrolyte interface, so that the impedance of the whole electrode can be reflected from the current output.

The current of the electrodes is measured based on the CV curves before Con A modification, after modification, and after regeneration. In the first round after Con A modification, as shown in Figure S11, a big current decrease can be seen from the oxidation peak ((I_A_-I_C_)/I_A_=29.2%) and reduction peak ((I_B_-I_D_)/I_B_=29.1%), verifying the increased resistance and the binding of Con A molecule on the electrode.

In the second round, a clear recovery of the current when 2.5 pM glucose is added and detected, verifying that the decreased resistance and the release of glucose. Thus, the reversibility of the sensing mechanism can be supported. Moreover, after the second round, the CV curve of the regenerated electrode was highly consistent with that of the original unmodified electrode. Only a very small current decrease ((I_initial_-I_final_)/I_initial_) can be seen from the oxidation peak ((I_A_-I_E_)/I_A_=4.2%) and reduction peak ((I_B_-I_F_)/I_B_=0.04%). This proves that not all of the Con A molecules can be completely removed from the electrode surface.

After 25 rounds reversible detection, the recovery of the electrode can be evaluated by the current decrease from the oxidation peak ((I_A_-I_G_)/I_A_=16%) and reduction peak ((I_B_-I_H_)/I_B_=26.4%). This further indicates that during the reversible detection process, Con A molecules cannot be completely removed from the electrode surface.

However, based on the photocurrent optimization from the previous part of the manuscript, even though the Con A molecules cannot be completely removed, the resistance effect they produce can be negligible for the enhanced photocurrent by the nanocomposites, thus enabling reversible detections.

**Figure S12** UV-visible (UV-vis) absorption spectroscopy measurements of different electrodes during 2 rounds glucose detection after 2.5 pM glucose is added and detected.

After Con A modification and glucose detection, the the Ag NP@Au NCs/Con A/ITO electrode and the Ag NP@Au NCs/Con A/glucose/ITO electrode are further used for UV-vis detection. Based on Figure S12, the UV-vis spectrum intensity decreases in the first two rounds when Con A is immobilized on the working electrode. This is because Con A molecules on the electrode surface block light transmission through the electrode during UV-vis measurements. After glucose detection, the intensity increases and almost recovers to the original value observed before Con A modification, supporting Con A removal and demonstrating the feasibility of reversible detection. This result supports the mechanism mentioned above in Figure 4a.

**Figure S13** Stability study from photocurrent-time (I-t) curves at 500 mV in 0.01 M PBS (pH=7.4) under continuous illumination for 1000 seconds.

**IV) References**

[1].	Hu, J., et al., *Highly sensitive and specific detection of human papillomavirus type 16 using CRISPR/Cas12a assay coupled with an enhanced single nanoparticle dark-field microscopy imaging technique.* Talanta, 2024. **278**: p. 126449.

[2].	Zhou, J., et al., *A Galvanic Exchange Process Visualized on Single Silver Nanoparticles Via Dark-Field Microscopy Imaging.* Nanoscale, 2018. **10**(26): p. 12805-12812.

[3].	Feng, W., et al., *Inconspicuous Reactions Identified by Improved Precision of Plasmonic Scattering Dark-Field Microscopy Imaging Using Silver Shell-Isolated Nanoparticles as Internal References.* Analytical Chemistry, 2019. **91**(4): p. 3002-3008.

[4].	Ouyang, T., et al., *Direct Excitation Transfer in Plasmonic Metal-Chalcopyrite-Hybrids: Insights from Single Particle Line Shape Analysis.* ACS Nano, 2024. **18**(32): p. 21565-21575.

[5].	Pang, J., et al., *Structural Change of a Single Ag Nanoparticle Observed by Dark-field Microspectroscopy.* ChemPhysChem, 2018. **19**(8): p. 954-958.

[6].	Oprica, L., et al., *Citrate-silver Nanoparticles and Their Impact on Some Environmental Beneficial Fungi.* Saudi Journal of Biological Sciences, 2020. **27**(12): p. 3365-3375.

[7].	Salimi, S., et al., *Rapid Quantification of Virus-Like Particles via Gold Nanoparticle Sensors and Dark-Field Differential Dynamic Microscopy.* ACS Sensors, 2025. **10**(10): p. 7712-7725.

[8].	Long, J., et al., *Detection of Aldehyde Compounds in Daily and Industrial Supplies by Colourimetry and Dark-field Microscope Imaging.* Sensors and Actuators B: Chemical, 2025. **439**: p. 137838.

[9].	Fukuzumi, N., et al., *Detection of C-reactive protein using single cluster analysis of gold nanoparticle aggregates using a dark-field microscope equipped with a smartphone.* Sensors & Diagnostics, 2025. **4**(2): p. 159-165.

[10].	Li, H., et al., *Resolving Artifacts and Improving the Detection Limit in Circular Differential Scattering Measurement of Chiral and Achiral Gold Nanorods.* ACS Nano, 2025. **19**(3): p. 3635-3644.

[11].	Zhao, Y., et al., *Dark-Field Microscopic Real-Time Monitoring the Growth of Au on Cu_2_O Nanocubes for Ultra-sensitive Glucose Detection.* Analytica Chimica Acta, 2021. **1162**: p. 338503.

[12].	Zheng, L., et al., *Hyperspectral Dark-Field MMicroscopy for Pathogen Detection Based on Spectral Angle Mapping.* Sensors and Actuators B: Chemical, 2022. **367**: p. 132042.

[13].	Xie, Y. F., et al., *Metal-Mediated Gold Nanospheres Assembled for Dark-Field Microscopy Imaging Scatterometry.* Talanta, 2019. **201**: p. 280-285.

[14].	Zhang, Q., et al., *Plasmonic Biosensor for the Highly Sensitive Detection of MicroRNA-21 via the Chemical Etching of Gold Nanorods under a Dark-Field Microscope.* Biosensors and Bioelectronics, 2022. **201**: p. 113942.

[15].	Moradi Kashkooli, F., et al., *Low-Intensity Pulsed Ultrasound Enhances Uptake of Doxorubicin-Loaded Gold Nanoparticles in Cancer Cells.* Ultrasonics Sonochemistry, 2025. **120**: p. 107417.

[16].	Lin, D., et al., *A Reversible Photoelectrochemical Microsensor for Dynamically Monitoring Sulfur Dioxide in the Epileptic Brain.* Chemical Science, 2024. **15**(13): p. 4824-4832.

[17].	Hu, J., et al., *A Photoelectrochemical Retinomorphic Synapse.* Advanced Materials, 2024. **36**(38): p. 2405887.

[18].	Xue, J., et al., *Bioinspired Iontronic Gas Sensor for Selective CO_2_ Sensing via Reversible Lysine Carbamylation.* ACS Sensors, 2025. **10**(10): p. 7538-7548.

[19].	Kim, K. B., et al., *Highly Selective and Reversible Detection of Simulated Breath Hydrogen Sulfide Using Fe-Doped CuO Hollow Spheres: Enhanced Surface Redox Reaction by Multi-Valent Catalysts.* Small, 2024. **20**(31): p. 2308963.

[20].	Aykanat, A., et al., *Bimetallic Two-Dimensional Metal–Organic Frameworks for the Chemiresistive Detection of Carbon Monoxide.* Angewandte Chemie International Edition, 2022. **61**(6): p. e202113665.

[21].	Liu, Z., et al., *A Reversible Fluorescent Probe for Real-Time Quantitative Monitoring of Cellular Glutathione.* Angewandte Chemie International Edition, 2017. **56**(21): p. 5812-5816.

[22].	Enbanathan, S., et al., *A New Fast-Responding Fluorimetric “Turn-on” Sensor based on Benzothiazole-Phenanthridine for the Sensitive, Selective, and Reversible Detection of Cu^2+^ in Real Water Samples and Its Use in Bio-Imaging.* Dyes and Pigments, 2022. **205**: p. 110514.

[23].	Lu, Q., et al., *Hydrophobicity Regulation of Energy Acceptors Confined in Mesoporous Silica Enabled Reversible Activation of Optogenetics for Closed-Loop Glycemic Control.* Journal of the American Chemical Society, 2023. **145**(10): p. 5941-5951.

[24].	Zhu, J., et al., *Background-Free and Reversible Upconversion Hydrogel Sensing Platform for Visual Monitoring of Sulfite.* Analytical Chemistry, 2024. **96**(6): p. 2711-2718.

[25].	Sharma, V., S. Savita, and G.K. Patra, *A Highly Sensitive Triazole-Based Perfectly Water Soluble Novel Bis-Schiff Base Reversible Fluorescent-Colorimetric Chemosensor for Fast Detection of Pb^2+^ Ions.* RSC Advances, 2024. **14**(5): p. 3289-3303.

[26].	Ananth, A., et al., *Reversible Immobilization of Proteins in Sensors and Solid-State Nanopores.* Small, 2018. **14**(18): p. 1703357.

[27].	Hühn, J., et al., *Selected Standard Protocols for the Synthesis, Phase Transfer, and Characterization of Inorganic Colloidal Nanoparticles.* Chemistry of Materials, 2017. **29**(1): p. 399-461.

[28].	Alnoush, W., R. Black, and D. Higgins, *Judicious Selection, Validation, and Use of Reference Electrodes for In Situ and Operando Electrocatalysis Studies.* Chem Catalysis, 2021. **1**(5): p. 997-1013.

[29].	Probst, D., I. Lee, and K. Sode, *The Development of Micro–Sized Enzyme Sensor Based on Direct Electron Transfer Type Open Circuit Potential Sensing Principle.* Electrochimica Acta, 2022. **426**: p. 140798.

[30].	Dadafarin, H., E. Konkov, and S. Omanovic, *Electrochemical Functionalization of a 316L Stainless Steel Surface with a 11-mercaptoundecanoic Acid Monolayer: Stability Studies.* International Journal of Electrochemical Science, 2013. **8**(1): p. 369-389.

[31].	Zhu, X., B. Li, and X. Liu, *Photoelectrochemical Sensing Hela Cell Mitochondria Released Superoxide Anion Using A Novel Heterojunction Formed by Dispersing a Wheel-Shaped Polyoxometalate on α-Fe_2_O_3_ Nanoarray.* Sensors and Actuators B: Chemical, 2025. **444**: p. 138324.

[32].	Li, H. J., et al., *Non-Invasive Ultrasensitive Photoelectrochemical Detection of Cancer Cell-Secreted H₂O₂ Using Hierarchical Silicon Nanowire/Co-MOF Core-Shell Nanoarrays.* Sensors and Actuators B: Chemical, 2026. **446**: p. 138700.
